# Liver Transplantation for Hepatocellular Carcinoma in Patients with Inherited Metabolic Liver Diseases: A Single-Center Analysis

**DOI:** 10.5152/tjg.2023.22679

**Published:** 2023-12-01

**Authors:** İbrahim Umar Garzali, Abdirahman Sakulen Hargura, Volkan İnce, Fatma İlknur Varol, Brian I. Carr, Sezai Yılmaz

**Affiliations:** 1 Department of Surgery, İnönü University, Liver Transplantation Institute, Malatya, Turkey; 2 Department of Pediatric Gastroenterology, İnönü University Faculty of Medicine, Malatya, Turkey

**Keywords:** Liver transplantation, hepatocellular carcinoma, inherited metabolic liver diseases, progressive familial intrahepatic cholestasis

## Abstract

**Background/Aims:**

Liver transplantation is an acceptable treatment for some selected hepatocellular carcinoma. We report our experience of 6 patients with liver transplantation for hepatocellular carcinoma with background inherited metabolic disease.

**Materials and Methods:**

This is a single-center retrospective, descriptive study. Consecutive patients who underwent liver transplantation for hepatocellular carcinoma with background inherited metabolic disease were included in the study. The record of the patients was accessed, and the following data were extracted: sociodemographic variables, type of metabolic disease, date of liver transplantation, tumor characteristics, laboratory parameters, Model for End-Stage Liver Disease score, immediate- and long-term outcome after transplantation, disease-free survival, and overall survival. Data were analyzed using Statistical Package for the Social Sciences version 25.0.

**Results:**

Six patients received liver transplantation for hepatocellular carcinoma with background inherited metabolic liver disease. The median age was 4.5 years. The median Model for End-Stage Liver Disease score was 29.30. The median maximum tumor diameter was 2.15 cm. Three patients had multiple tumor nodules. Half of the patients had microvascular invasion. Four of the patients had a moderately differentiated tumor. Progressive familial intrahepatic cholestasis type II is the commonest inherited metabolic disease seen in 3 patients. Median follow-up is 46.1 months. Half of the patients are currently more than 5 years post liver transplantation with no features of recurrence. The estimated survival rates at 1, 3, and 5 years are 100%, 83.3%, and 83.3%, respectively.

**Conclusion:**

Liver transplant for these categories of patients is associated with good disease-free and overall survival, even in the presence of some seemingly poor prognostic features.

**Main Points**

- Inherited metabolic liver disease can progress to cause liver cirrhosis and hepatocellular carcinoma (HCC).
- Liver transplant for HCC with background inherited metabolic liver disease has a good outcome.
- Presence of poor prognostic features in HCC may not be associated with poor outcome in this group of patients.

## Introduction

Hepatocellular carcinoma (HCC) is the sixth most common cancer, with 905 677 new cases diagnosed in 2020 globally.^1^ It is the third leading cause of cancer death globally, with 830 180 deaths reported in 2020^1^, and represents 75%-85% of all liver cancer cases.^1^

There are multiple factors that are associated with increased risk for the development of HCC, and most of them share a common characteristic of injury to the liver parenchyma, resulting in cirrhosis.^2, 3^ The most common global cause of cirrhosis is chronic infection with hepatitis B virus (HBV) or hepatitis C virus (HCV).^2, 3^ In some studies, up to 50% of cases were attributed to chronic HBV and 20% of cases were attributed to chronic HCV infection.^4^ However, patients with chronic HBV infection are at risk for HCC even in the absence of cirrhosis. Patients with cirrhosis from any etiology are also at risk for developing HCC. Recent estimations revealed that up to 2.5% of individuals with cirrhosis will develop HCC annually.^5^ In contrast to adults, pediatric HCC is a rare disease, and it is only the second most common malignant liver lesion in children.^6, 7^ It is second to hepatoblastoma in pediatric patients. The characteristics of pediatric HCC also differ from adult HCC in etiology, tumor biology, and association with hepatic cirrhosis.^6, 7^ In this group of patients, the common risk factors for HCC include progressive familial intrahepatic cholestasis, glycogen storage disease, perinatal HBV, hepatorenal tyrosinemia, Alagille syndrome, and congenital portosystemic shunts.^6, 7^ Fibro-lamellar variant of HCC is relatively commoner in pediatric and constitutes 15%-25% of pediatric HCC compared to 5% in adults. In pediatric patients, HCC with no history of background liver disease is seen in 70% of patients, while 30% of patients have associated background liver disease.^6, 7^

Inherited metabolic diseases are associated with liver dysfunction and injury.^8^ The liver injury is usually precipitated by the accumulation of a toxic metabolite within the liver with associated chronic liver injury, which may progress to cirrhosis and subsequently to HCC.^9, 10^ The “toxic metabolite” theory of liver injury is exemplified by the accumulation of alpha 1 anti-trypsin within the endoplasmic reticulum of hepatocytes with resultant hepatocyte injury in patients with alpha 1 anti-trypsin deficiency.^11^ Non-alcoholic steatohepatitis, a spectrum of liver disease seen in patients with metabolic syndrome, also progresses to cirrhosis and even HCC.^12^ Inherited metabolic diseases can progress to HCC without passing through the cirrhotic stage of liver injury.^11^

Liver transplantation (LT) is considered the best treatment for early-stage HCC associated with cirrhosis. This is because it treats both the tumor and the underlying liver disease. Currently, HCC accounts for 15%-50% of all LT performed in most centers. Tumor recurrence following LT is estimated to be approximately 8%-20% in most studies.^13^

Recurrence after LT for HCC with background inherited metabolic liver diseases was said to be low compared to the patients who received transplant for HCC without inherited metabolic diseases.^14^

We report in this study our experience of 6 patients with LT for HCC with background inherited metabolic disease.

## MATERIALS AND METHODS

This is a single-center retrospective, descriptive study conducted in the Liver Transplantation Institute, Malatya, Turkey. Patients who underwent LT were analyzed for possible inclusion into the study.

### Diagnosis of Hepatocellular Carcinoma

Dynamic contrast-enhanced computed tomography or magnetic resonance imaging was used in diagnosing HCC. Diagnosis is confirmed by a distinct homogeneous or inhomogeneous “wash-in” enhancement in the arterial phase and a “wash-out” enhancement in the portal venous phase. For hepatic lesions without typical imaging characteristics, image-guided liver biopsy is performed for definitive pathologic diagnosis.

### Inclusion Criteria

Consecutive patients who underwent LT for HCC with background inherited metabolic disease were included in the study.

### Exclusion Criteria

1. All patients who received LT for indications other than HCC.
2. Patients who had LT for HCC but without metabolic liver disease.
3. Patients undergoing re-transplantation.

### Ethical Approval

Approval was obtained from the institutional review board of Liver Transplantation Institute, Malatya, Turkey (Approval No: 2022/3916, Date: 04-10-2022). Consent was obtained from the patients included in this study. In cases where the patient is below 18 years of age, consent was obtained from the guardian.

### Data Collection and Data Analysis

The medical record of the patients fulfilling the inclusion criteria was accessed, and the following data were extracted: sociodemographic variables, type of metabolic disease, date of LT, criteria for patient selection, macroscopic or microscopic tumor characteristics, laboratory parameters, Child’s score, Model for End-Stage Liver Disease (MELD)/Pediatric End-Stage Liver Disease (PELD) score, type of transplant, immediate- and long-term outcome after transplantation, disease-free survival (DFS) and overall survival (OS). Data were analyzed using Statistical Package for the Social Sciences version 25.0 (IBM Corp.; Armonk, NY, USA). Qualitative variables were expressed as ratio and percentage, while quantitative variables were presented as mean, median, range, and SD.

## Results

**Figure 1.**
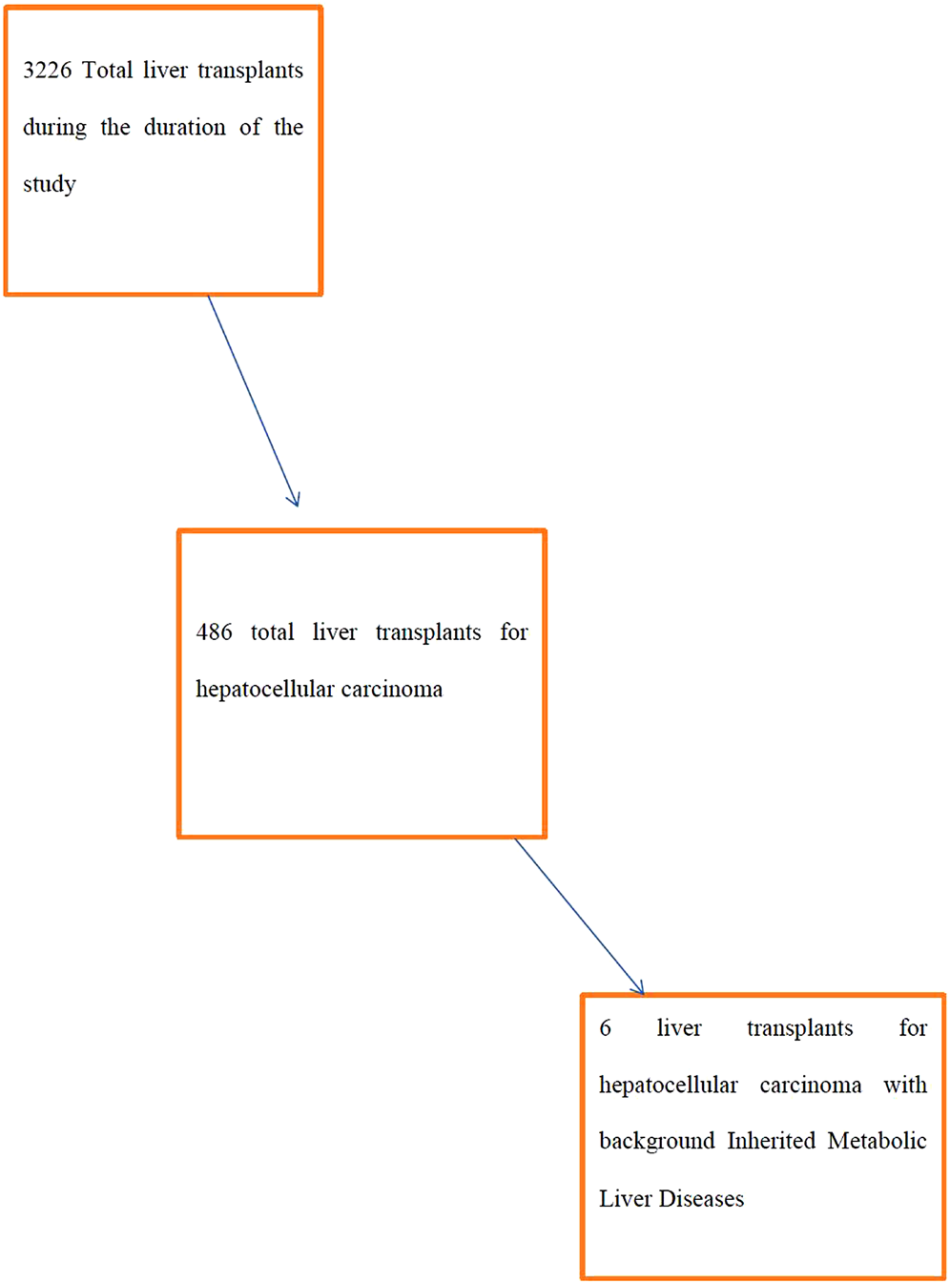
Diagrammatic representation of all patients that had liver transplant and those that were included in the study.

From April 2006 to May 2022, a total of 3226 LT were conducted in the Liver transplantation Institue, Malatya, Turkey. Out of these, 486 (15%) of them were transplanted for HCC. Only 6 (1.23%) of patients of the HCC group had background inherited metabolic liver disease. Figure 1 shows the diagrammatic representation of all patients who had LT and those that were included in the study.

### Sociodemographic and Baseline Clinical Characteristics

The median age of the patients included was 4.5 years with the range from 2 to 59 years. The male to female ratio is 2:1 with a mean body mass index of 18.47 ± 1.64. Child’s class C was seen in 4 patients, while Child’s class A and B were seen in 1 patient each. The median model for end stage liver disease/Pediatric End-Stage Liver Disease (MELD/PELD) score is 29.30 with a range of 6.0-37.0. The median total bilirubin was 16.05 mg/dL with a range of 0.43-33.83 mg/dL. The median aspartate aminotransferase (AST) was 165.5U/L with a median of 22-589 U/L. The median alanine transaminase (ALT) was 51U/L with a range of 18-289 U/L.

### Tumor Characteristics

The tumor characteristics among the patients showed a median maximum tumor diameter of 2.15 cm with a range of 0.1-4.0 cm. Three of the 6 patients had a single tumor nodule, while the others had multiple tumor nodules. No macroscopic vascular invasion was seen, but half of the patients had microvascular invasion. Four of the patients had a moderately differentiated tumor, while 2 had a well-differentiated tumor. None had a poorly differentiated tumor (Table 1).

### Background Inherited Metabolic Disease

Progressive familial intrahepatic cholestasis type II (PFIC-II) is the commonest inherited metabolic disease seen in this patient group, and it affected 3 patients (50%). A single patient had progressive familial intrahepatic cholestasis type I (PFIC-I). Hereditary tyrosinemia was seen in 1 patient, while Wilson’s disease was found in 1 patient (Table 2).

### Type of Transplantation and the Criteria for Patient Selection

Living donor LT was done for 5 patients, while only 1 of the patients received cadaveric graft for transplantation. Milan and Malatya criteria were applicable to 5 patients, while 1 patient was within extended Malatya criteria.

### Recurrence and Survival After Transplant

Median follow-up is 46.1 months with a range of 19.08-159.6 months. All but 1 patient are alive and disease free at the time of last follow-up. Half of the patients are currently more than 5 years post LT with no features of recurrence. Two of the remaining patients are more than 2 years post transplantation. The single mortality in the group occurred within 2 years of transplantation and was not related to recurrence. The patient developed chronic graft rejection which progressed to cause death.

## Discussion

Inherited metabolic liver disease constitutes about 3.3% of all indications for LT among all groups, but in the pediatric population, Rosencrantz et al^15^ reported that it constituted about 45% of the indications for LT. In our study, inherited metabolic liver disease makes up to 5.24% of all the patients who received LT within the period of study.

Hepatocellular carcinoma is responsible for 12.6% of all adult LT in the USA in 2020, while in Europe, it was responsible for 23% LT among all age groups.^16, 17^ Baumann et al^14^ revealed that HCC related to inherited metabolic liver diseases constitutes about 20.5% of all pediatric HCC patients who were registered in the European liver transplant registry. In our study, HCC was responsible for 15% of all LT, but HCC related to inherited metabolic liver disease was responsible for only 1.23% of the HCC patients receiving LT.

Progressive familial intrahepatic cholestasis type II is the commonest inherited metabolic disease seen in this patient group, and it affected 3 patients (50%). A single patient had PFIC-I. Hereditary tyrosinemia was seen in 1 patient, while Wilson’s disease was seen in the last patient. This is similar to the findings of Celik and Emiroglu^18^ in a study of 14 children who received LT for HCC with background inherited metabolic liver disease. In their study, they found that PFIC was responsible for 57.14% HCC with inherited liver diseases. In the study by Rosencrantz et al,^15^ there were 4 patients with incidental diagnosis of HCC in the explanted liver after transplantation for inherited metabolic liver disease, and 75% of the patients had inherited tyrosinemia while 1 had Wilson’s disease. Baumann et al^14^ and Ozçay et al^19^ also found that among patients with HCC and background inherited metabolic liver diseases, tyrosinemia was the most common disease with findings of 23.08% and 50%, respectively.

Median follow-up in our study population was 46.1 months with a range of 19.08-159.6 months. At the time of last follow-up, all patients but 1 were alive and disease free. Half of the patients in our study population are currently more than 5 years post LT with no features of recurrence, while 2 of the remaining patients are more than 2 years post transplantation. The estimated survival rates at 1 year, 3 years, and 5 years are 100%, 83.3%, and 83.3%, respectively. The survival rate in the patients is good, considering that up to 50% of our patients had microvascular invasion, 50% had multicentric tumor, and 50% had alpha feto protein of >200 at the time of transplantation. Our study has similar findings to Ozçay et al^19^ who reported no mortality or recurrence after a median follow-up of 18.8 months among patients who underwent LT for HCC with background inherited metabolic liver disease. This good outcome was also reflected in the comparative study by Baumann et al,^14^ who reported that patients with HCC with background inherited metabolic liver disease had a better immediate- and long-term outcome compared to patients who received LT for HCC not related to inherited metabolic liver disease. They attributed this difference to the possible close follow-up of patients diagnosed with inherited metabolic disease and thus the possibility of earlier detection of the HCC in this group of patients, since they were being monitored closely.

The main limitation of this study is the small number of patients who had LT for HCC with background inherited metabolic liver diseases. Multicenter and prospective studies are needed for more definitive results.

## Conclusion

Even though inherited metabolic liver diseases can progress to liver cirrhosis and HCC, LT for this category of patients is associated with good DFS and OS, even in the presence of some seemingly poor prognostic features.

## Figures and Tables

**Table 1.** Baseline Laboratory and Tumor Characteristics

	Parameter Value	Frequency
1	Maximum tumor diameter, median >5 cm, n (%)	
	Mean = 2.71 cm ± 1.55 cm, median (minimum–maximum) = 2.15 (0.1-4.0)	0 (0%)
2	MELD/PELD score, median (minimum–maximum)	
	Mean = 24.77 ± 11.51, median (minimum–maximum) = 29.30 (6.0-37.0)	
3	Child’s class	
			Class A	1
			Class B	1
	Class C	4
4	Number of tumor nodules	
			Single	3
	Multiple	3
5	Type of liver transplant	
			LDLT	5
	DDLT	1
6	Level of tumor differentiation	
			Well-differentiated tumor	2
			Moderately differentiated tumor	4
	Poorly differentiated tumor	0
7	Vascular invasion	
			No invasion	3
			Microvascular invasion	3
	Macrovascular invasion	0
8	AFP	
	Median (minimum–maximum)	
	1367 ng/mL (12.3-2598)	
	>200, n (%)	3 (50%)
9	Milan criteria	
			Within	5
	Beyond	1
10	Extended Malatya criteria	
			Within	5
	Beyond	1
11	GGT	
	>104 IU/mL	0

AFP, Alpha-fetoprotein; DDLT, deceased donor liver transplantation; GGT, gamma-glutamyl transferase; LDLT, living donor liver transplantation; MELD, Model for End-Stage Liver Disease; PELD, Pediatric End-Stage Liver Disease.

**Table 2.** Inherited Metabolic Diseases Seen in Patients

	Disease	Frequency
1	Progressive familial intrahepatic cholestasis type II	3
2	Progressive familial intrahepatic cholestasis type I	1
3	Tyrosinemia	1
4	Wilson’s disease	1
